# Identification of the Microbial Transformation Products of Secoisolariciresinol Using an Untargeted Metabolomics Approach and Evaluation of the Osteogenic Activities of the Metabolites

**DOI:** 10.3390/molecules28155742

**Published:** 2023-07-29

**Authors:** Wen-Xuan Yu, Hok-Him Tang, Jun-Jie Ye, Hui-Hui Xiao, Chung-Yan Lam, Tim-Fat Shum, Zhi-Kang Sun, Yuan-Zhen Li, Xin-Yu Zang, Wen-Chao Du, Jian-Ping Zhang, Tsz-Hung Kong, Li-Ping Zhou, Jia-Chi Chiou, Chun-Fai Kung, Kam-Wah Mok, Jing Hu, Man-Sau Wong

**Affiliations:** 1 Department of Food Science and Nutrition, The Hong Kong Polytechnic University, Hung Hom, Hong Kong, China; wxuanyu@polyu.edu.hk (W.-X.Y.); timothy-hh.tang@connect.polyu.hk (H.-H.T.); huihui.xiao@polyu.edu.hk (H.-H.X.); joyjai.lam@polyu.edu.hk (C.-Y.L.); tim-fat-perry.shum@connect.polyu.hk (T.-F.S.); tsz-hung.kong@polyu.edu.hk (T.-H.K.); jiachi.amber.chiou@polyu.edu.hk (J.-C.C.); man-sau.wong@polyu.edu.hk (M.-S.W.); 2 Increasepharm (Tianjin) Innovative Medicine Institute Limited, Tianjin 300382, China; yejunjie@ykrskj.com (J.-J.Y.); sunzhikang@ykrskj.com (Z.-K.S.); liyuanzhen@ykrskj.com (Y.-Z.L.); zangxinyu@ykrskj.com (X.-Y.Z.); 823119525@163.com (W.-C.D.); zhangjianping@ykrskj.com (J.-P.Z.); 3 State Key Laboratory of Chinese Medicine and Molecular Pharmacology (Incubation), Shenzhen Research Institute of the Hong Kong Polytechnic University, Shenzhen 518057, China; 4 Research Centre for Chinese Medicine Innovation, The Hong Kong Polytechnic University, Hung Hom, Hong Kong, China; lp.zhou@polyu.edu.hk; 5 Research Institute for Future Food, The Hong Kong Polytechnic University, Hung Hom, Hong Kong, China; 6 Increasepharm (HK) Limited, Hong Kong Science Park, Shatin, Hong Kong, China; angelakung@ykrskj.com

**Keywords:** secoisolariciresinol, microbial transformation, metabolites, osteogenic activities

## Abstract

Secoisolariciresinol (SECO) is one of the major lignans occurring in various grains, seeds, fruits, and vegetables. The gut microbiota plays an important role in the biotransformation of dietary lignans into enterolignans, which might exhibit more potent bioactivities than the precursor lignans. This study aimed to identify, synthesize, and evaluate the microbial metabolites of SECO and to develop efficient lead compounds from the metabolites for the treatment of osteoporosis. SECO was fermented with human gut microbiota in anaerobic or micro-aerobic environments at different time points. Samples derived from microbial transformation were analyzed using an untargeted metabolomics approach for metabolite identification. Nine metabolites were identified and synthesized. Their effects on cell viability, osteoblastic differentiation, and gene expression were examined. The results showed that five of the microbial metabolites exerted potential osteogenic effects similar to those of SECO or better. The results suggested that the enterolignans might account for the osteoporotic effects of SECO in vivo. Thus, the presence of the gut microbiota could offer a good way to form diverse enterolignans with bone-protective effects. The current study improves our understanding of the microbial transformation products of SECO and provides new approaches for new candidate identification in the treatment of osteoporosis.

## 1. Introduction

Osteoporosis is an age-related skeletal disorder characterized by low bone mass and deterioration of the bone microarchitecture, which make people more susceptible to fractures. Women are at higher risk of osteoporotic fractures due to their abruptly decreased estrogen levels after menopause, which accelerate bone loss in women [1]. Estrogen replacement therapy is currently used for the clinical treatment of osteoporosis [2]. However, its high price and unexpected adverse effects limited their application [3,4]. As a result, there is great demand for novel alternative treatment strategies for osteoporosis.

Lignans are recognized as phytoestrogens due to their steroid-analogous chemical structure, enabling them to activate estrogen receptors and modulate estrogen-dependent processes [5,6]. Secoisolariciresinol (SECO) is one of the predominant lignans occurring in various grains, seeds, fruits, and vegetables, such as flaxseed and sesame seeds [7,8]. Dietary lignans impart pharmacological effects due to their antioxidant [9,10], estrogenic/antiestrogenic [11], anti-aromatase [12], and anti-inflammatory [13] properties, thereby exhibiting multifaceted beneficial effects [14,15,16]. Accumulating evidence highlights the beneficial effects of a lignan-rich diet in maintaining bone mineral density and bone metabolism in postmenopausal women [17,18,19,20].

The bioavailability of dietary lignans is very limited, as they are seldom absorbed in the small intestine or excreted in urine [21,22,23]. However, a large fraction of them reach the colon and are metabolized by intestinal bacteria in a series of steps of hydrolysis, dehydroxylation, demethylation, and oxidation to form “mammalian” lignans, enterodiol, and enterolactone, which were first detected in the urine of humans and female rats fed with flaxseed [24,25]. Unlike natural lignans present in plants, these “mammalian” lignans carry phenolic hydroxy groups only in the meta position of the aromatic rings [26]. Numerous studies have indicated that the estrogen–receptor binding affinity and bioactivities of the “mammalian” lignans are more potent than those of plant lignans, thus emphasizing the role of gut microbiota in biotransformation [27,28,29].

Our previous studies demonstrated that a lignan-rich fraction of *Sambucus williamsii* Ramulus prevented bone loss and improved bone strength and bone microarchitecture in ovariectomized mice and rats [30,31]. However, the microbial transformation products, but not the precursor lignans, were detected in the plasma of the animals after drug administration. It is unknown whether microbial transformation products are potent in regard to potential bone protective effects [32]. Moreover, many previous studies reported on the biotransformation of SECO by gut microbiota, yielding enterodiol and enterolactone, while the intermediate metabolites have rarely been reported [33,34]. It would be interesting to investigate the pathway of SECO biotransformation by microflora and to explore novel enterolignans with potent osteogenic activities.

The present study aimed to identify the microbial metabolites of SECO produced by gut microbiota and to develop efficient lead compounds from SECO for the treatment of osteoporosis. The dynamic profile of SECO and the microbial fermentation metabolites were determined through an untargeted metabolomics approach using liquid chromatography–mass spectrometry (LCMS). The microbial transformation pathway of SECO was proposed based on the dynamic profile and chemical structures of the metabolites. The identified metabolites were synthesized or purchased. The effects of the identified metabolites on the cell viability, osteoblastic differentiation, and gene expression of osteogenic markers were further evaluated in murine pre-osteoblast MC3T3-E1 cells and human osteoblast-like MG-63 cells.

## 2. Results

### 2.1. Multi-Variance Analysis

**Figure 1:**
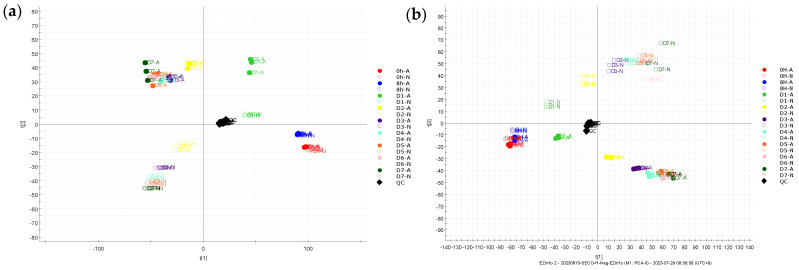
PCA plot of the metabolic profiles of fecal samples incubated with SECO in (**a**) the +ESI mode and (**b**) −ESI mode. A: micro-aerobic conditions; N: anaerobic conditions.

**Figure 2:**
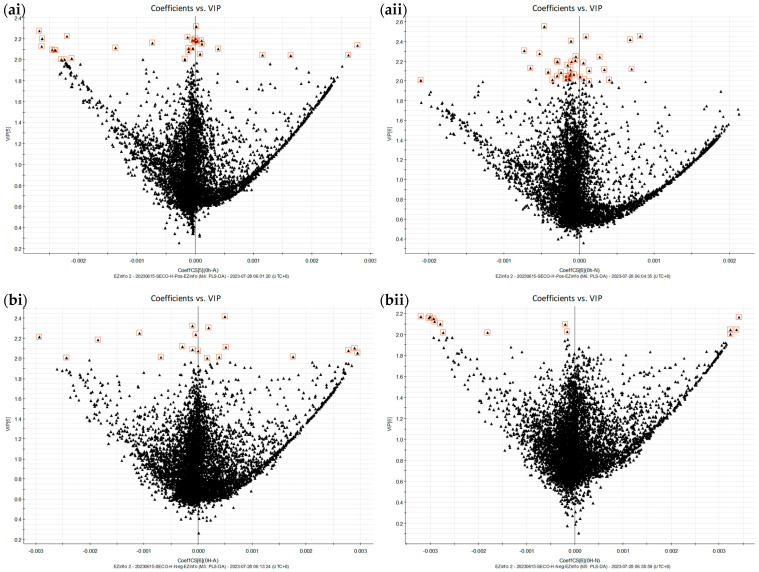
PLS-DA VIP vs. coefficient plot of the metabolic profiles of fecal samples incubated with SECO in the (**a**) +ESI mode and (**b**) −ESI mode. (**i**): Micro-aerobic conditions; (**ii**): anaerobic conditions.

The prediction of the metabolites of SECO was based on reasonable biotransformation patterns observed in SECO and its known end products, enterodiol and enterolactone [35], as well as biotransformations suggested by metabolism prediction sources such as BioTransformer 3.0 [36]. Figure 1 and Figure 2 show the results of the multivariate analysis, which revealed that samples with similar metabolic profiles were clustered together on the principal component analysis (PCA) plot, while samples with dissimilar metabolic profiles were located farther apart. Furthermore, the quality control (QC) samples demonstrated a high degree of aggregation in both the positive and negative modes, indicating good stability throughout the analytical run. Nevertheless, the clustering of all the samples within groups and the separation between groups suggested significant differences in the metabolites obtained at different time points. As the PCA plot (Figure 1) shows, the samples were clearly separated based on their different incubation time points in the first component, which means that the most representative difference was caused by the incubation time. On the other hand, a distinct separation could be observed based on the incubation conditions in the second component, and the difference between anaerobic and microaerobic conditions increased over time. This implied that the variation in the metabolic profile was significantly influenced by not only the incubation time but also the incubation conditions. Variable importance projection (VIP) plots (Figure 2) were employed to investigate the metabolites that contributed to the variation at each time point. Metabolites with VIP scores greater than 2.0 were considered to provide significant contributions to the model. In the positive ESI mode, 257 and 219 metabolites had high VIP values under micro-aerobic and anaerobic conditions, respectively, while in the negative mode, 209 and 192 metabolites, respectively, showed high VIP values. Importantly, the majority of the proposed metabolites (7 out of 9) were present among the metabolites with high VIP values, which indicates that these metabolites are the major contributors to the observed differences in the models. 

### 2.2. Metabolite Identification

**Figure 3:**
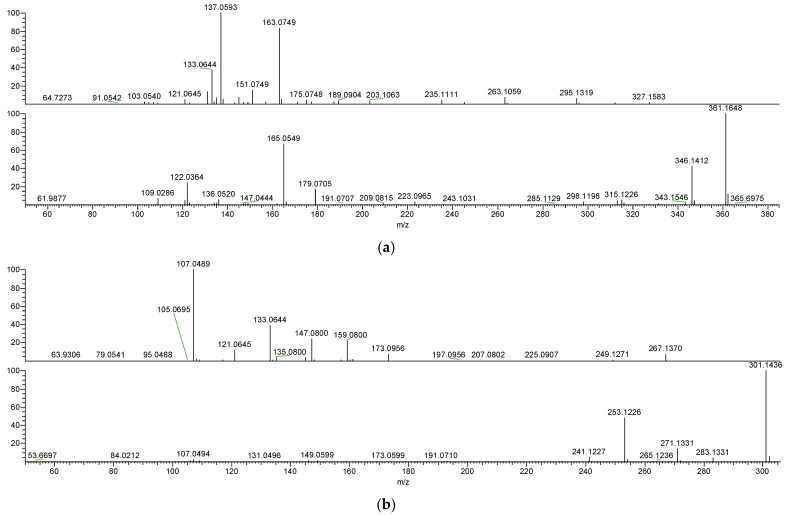

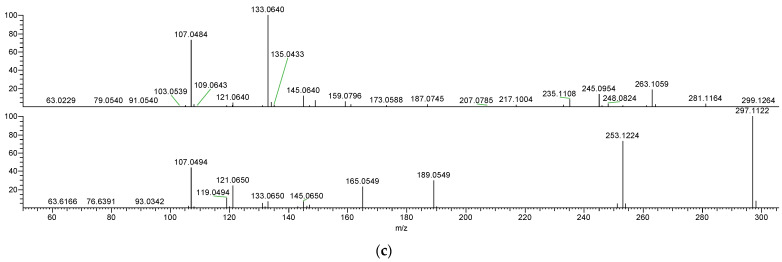
MS2 spectra of (**a**) SECO, (**b**) enterodiol, and (**c**) enterolactone in the +ESI mode and −ESI mode.

A series of calculations were performed to determine the mass to charge ratio of each proposed metabolite, taking into account its various adduct forms, and the result was subsequently extracted from the total ion chromatogram (TIC) of the QC. Then, the MS2 spectra were examined and compared with the SECO, enterodiol, and enterolactone standards to evaluate their similarity (Figure 3). Metabolites with a high degree of similarity to the standards were considered to have a higher possibility of being intermediates in the SECO metabolic pathway. It is important to note that while some metabolites were detectable through MS1 scanning, their MS2 fragments were undetectable. A comprehensive overview is shown in Table 1, which summarizes the retention time (Rt), mass to charge ratio, adduct form, and similar MS2 fragments.

### 2.3. Proposed Metabolic Pathway

**Figure 4:**
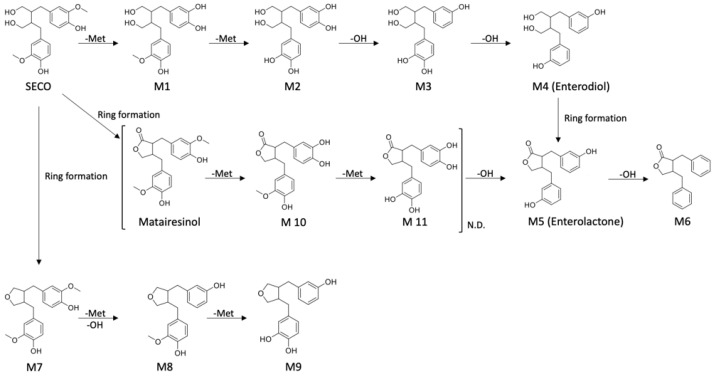
Proposed microbial transformation pathway. −Met and −OH indicate demethylation and dehydroxylation, respectively.

The proposed SECO metabolic pathway is illustrated in Figure 4. The demethylation of SECO was the first step towards the formation of enterolignans, and therefore, metabolites **M1** and **M2** were generated. **M2** underwent a series of dehydroxylation reactions to convert it into **M3** and enterodiol. After that, enterodiol (**M4**) was converted into enterolactone (**M5**) with the closure of the lactone ring. This is not the only pathway available. It has been reported that lactones can be generated directly from SECO, yielding matairesinol [37]. Following matairesinol, **M10**, **M11**, and enterolactone were similarly generated through the above-described demethylation and dehydroxylation. However, neither matairesinol, **M10**, nor **M11** was detected during analysis. It is possible that the abundance of metabolites was below the limit of detection (LOD) or the actual metabolic pathway differs from our initial suggestion. It seems that the generation of enterolactone was not necessarily the last step of transformation. M6 could be produced through further dehydroxylation. In addition, SECO was also transformed into cyclic ethers through the same series of reactions, yielding **M7**, **M8**, and **M9**.

### 2.4. Biotransformation of SECO over Time

**Figure 5:**
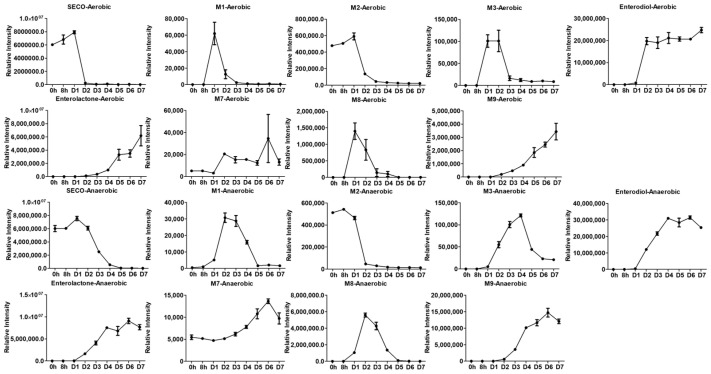
Time course of SECO transformation in human gut microbiota culture.

The relative intensity change over time is shown in Figure 5. Unexpectedly, the relative intensity on day 1 of SECO as a precursor was observed to be higher than that at 0 h and 8 h. In addition, the abundance of SECO sharply decreased after incubation for one day, especially under micro-aerobic conditions. It is highly possible that the absorption of SECO was completed at approximately 24 h, and then it was rapidly metabolized. The abundance of **M1**, the initial metabolic product of SECO, began to increase on day 1, and this observation supports the hypothesis mentioned above. In contrast to **M1**, **M2** was detected at 0 h and 8 h. A slight increase in the abundance of **M2** was observed on day 1, followed by a drop on day 2, which was similar to the pattern observed for **M1**. One of the possible reasons for this is that the metabolic rate of change from SECO to **M2** is very fast, so that M1 is metabolized immediately once it is generated, which is supported by the low abundance of M1 compared with SCEO and **M2**. As a result, M1 was almost undetectable at 0 h and 8 h, except on day 1, which had the highest concentration. **M3** began to increase on day 1 and reached the highest abundance on the day that its precursor, **M2**, started to drop. The relative intensity of the metabolic end products, enterodiol and enterolactone, continued to increase from day 1 until the end of the experiment.

Additionally, the cyclic ether pathway (**M7**, **M8**, and **M9**) was proposed and detected together with the enterolactone pathway. Unlike the changes in abundance observed in **M1**, **M7** showed a gradual increase over time, with a small drop on 7th day. The abundance of **M8** was observed to increase in parallel with **M7**. However, the increase in **M8** was a sharp instead of slow growth, and then it kept decreasing for 3 days. **M9**, as the end product of this metabolic pathway, showed a very similar pattern to enterolactone, which is also the end product of SECO, but the abundance of **M9** was around 50% lower than that of enterolactone. In summary, **M9** is another metabolic pathway of SECO, but it differed from the expected metabolic pathway. One of the possibilities is that this is not the preferable pathway, resulting in a low metabolic rate and low abundance of metabolites. Additionally, some intermediates may not be detectable because of their low abundance.

### 2.5. Osteogenic Effects of SECO and Microbial Transformation Products

**Figure 6:**
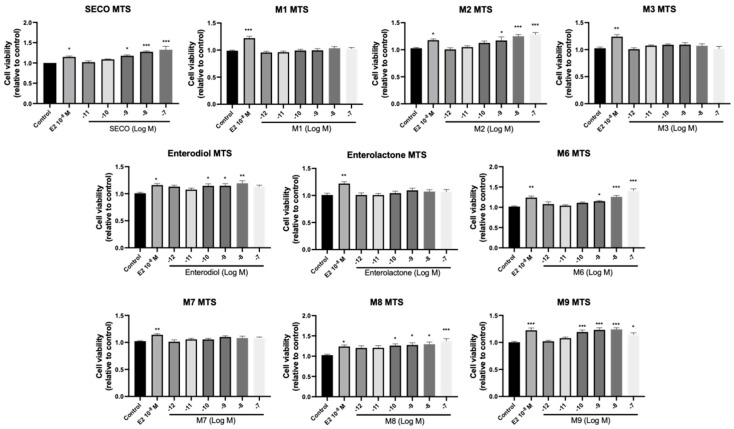
Effects of SECO and identified metabolites on cell viability of MC3T3-E1 cells. Cells were treated with vehicle, E_2_ (10^−8^ M), and different compounds (10^−12^−10^−7^ M) in phenol red-free medium for 24 h. Cell viability was measured via MTS assay. Data are shown as mean ± SEM and analyzed using one-way ANOVA, followed by Tukey’s multiple comparison test. * *p* < 0.05, ** *p* < 0.01, *** *p* < 0.001 vs. control (*n* = 6).

**Figure 7:**
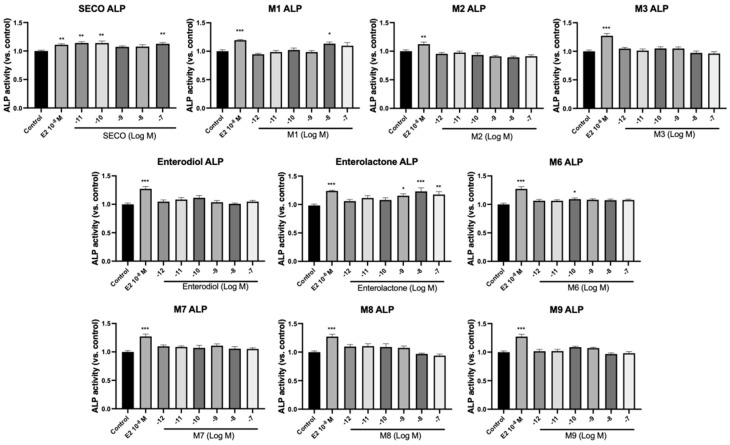
Effects of SECO and identified metabolites on cell differentiation of MC3T3-E1 cells. Cells were treated with vehicle, E_2_ (10^−8^ M), and different compounds (10^−12^−10^−7^ M) in osteogenic medium for 7 days. Cell differentiation was measured via ALP activity assay. Data are shown as mean ± SEM and analyzed using one-way ANOVA, followed by Tukey’s multiple comparison test. * *p* < 0.05, ** *p* < 0.01, *** *p* < 0.001 vs. control (*n* = 6).

ALP is produced by osteoblasts and plays important roles in osteoid formation and mineralization, therefore acting as a marker for bone formation [38]. In order to understand the osteogenic effects of microbial transformation products, osteoblastic cell viability and ALP activity were examined in pre-osteoblast MC3T3-E1 cells. E_2_ (10^−^^8^ M) was selected as a positive control due to its recognized anabolic effects on bone cells through estrogen-dependent pathways [39]. As shown in Figure 6 and Figure 7, SECO significantly increased the cell viability of MC3T3-E1 cells at 10^−^^9^ to 10^−^^7^ M (*p* < 0.05 vs. control) and cell differentiation at 10^−^^11^, 10^−^^10^, and 10^−^^7^ M (*p* < 0.01 vs. control). For the metabolites, **M2**, enterodiol, **M8**, and **M9** dose-dependently improved cell viability with lowest concentrations of 10^−^^9^ (*p* < 0.05 vs. control), 10^−^^10^ (*p* < 0.05 vs. control), 10^−^^10^ (*p* < 0.05 vs. control), and 10^−^^10^ M (*p* < 0.001 vs. control), respectively. However, they did not show significant effects on ALP activity. In contrast, **M1** at 10^−^^8^ M (*p* < 0.05 vs. control) and enterolactone at 10^−^^9^ M (*p* < 0.05 vs. control) only markedly elevated ALP activity. **M6**, which removed all the phenolic hydroxy groups after a series of dehydroxylation, showed significant effects on both cell viability (*p* < 0.05 vs. control) and osteogenic cell differentiation (*p* < 0.05 vs. control).

### 2.6. Effects of Microbial Transformation Products on the Gene Expression of Bone Markers

To further compare the effects of the identified metabolites on the interaction between osteoblasts and osteoclasts, the gene expression levels of OPG and RANKL were evaluated in human osteoblast-like MG-63 cells. As shown in Table 2, **M2**, enterolactone, **M6**, **M8**, and **M9** significantly upregulated the gene expression of OPG (*p* < 0.05 vs. control) while suppressing that of RANKL (*p* < 0.05 vs. control), resulting in an increased ratio of OPG/RANKL. On the contrary, enterodiol at 10^−^^12^ M showed a significant reduction in RANKL gene expression (*p* < 0.01 vs. control) and an increase in the ratio of OPG/RANKL (*p* < 0.01 vs. control). These results suggested that **M2**, enterolactone, **M6**, **M8**, and **M9** might regulate bone remodeling by increasing osteoblastic bone formation and suppressing osteoclastic bone resorption, while enterodiol only exerts anti-resorption effects.

## 3. Discussion

The role of gut microbiota in the transformation of lignans is well-recognized. Enterolignans generated from the biotransformation of dietary lignans have been linked to the prevention of cardiovascular disease, as well as prostate and breast cancer. However, their effects on bone health are rarely reported [40]. The purpose of this study was to characterize the microbial transformation products of SECO using an untargeted metabolomics approach, to synthesize the identified metabolites, and to further evaluate the effects of the metabolites on osteogenesis in osteoblastic cells.

In the present study, SECO was fermented with human fecal gut microbiota in vitro. The in vitro approach allows one to determine the metabolic pattern of gut microbiota by analyzing samples collected at different incubation time points. The results were in line with the expected metabolic pattern in that SECO underwent demethylation and dehydroxylation, yielding the intermediates **M1**, **M2**, **M3**, and enterodiol, which is finally transformed into enterolactone, as reported in previous studies [41]. The results herein support the notion that the second metabolic pathway of SECO generates **M7**, **M8**, and M9 through the formation of cyclic ethers [33]. However, the matairesinol pathway (matairesinol, **M10**, and **M11**) was not detected in the present work. In agreement with the current findings, it was suggested that matairesinol was not always detected as an intermediate of SECO [37]. One of the possibilities is that this is not the preferable pathway, resulting in a low metabolic rate and low abundance of metabolites. Additionally, some intermediates may not be detectable because of their low abundance. It has been suggested that the addition of formic acid might stabilize the metabolites in microbial culture samples [33].

The present study certainly supports the notion of the biotransformation of SECO into enterolignans by gut microbiota. Enterolignan production by the intestinal microbiota is quite extensive. As reported in the previous studies, SECO can be converted into enterodiol and enterolactone by a number of intestinal bacteria capable of performing demethylation, dehydroxylation, and dehydrogenation [42,43,44]. *Peptostreptococcus productus*, *Eubacterium limosum*, and *Clostridium methoxybenzovorans* are able to demethylate the lignans [45]. *Eggerthella lenta* is responsible for the dehydroxylation of dihydroxy-enterodiol (**M2**) to produce enterodiol [42,46]. Several *Clostridia* and *Ruminococcus* spp. strains have also been described in the dehydrogenation of enterodiol into enterolactone. It is worth noting that the above gut microbes involved in biotransformation are anaerobes. To ensure the bioactivities of these strains, the microbial culture was incubated in an anaerobic environment, as previously reported [33,47]. However, a recent study showed that three strains of *Lactobacillus* could yield both enterodiol and enterolactone from flax extracts [48]. Moreover, some *Lactobacillus* strains showed strong substrate specificity for SECO [48,49]. These findings suggest that microaerobes might play an important role in lignan fermentation. In support of this hypothesis, the results showed that the metabolites detected in the anaerobic culture were also detected in the microaerobic culture of SECO. Nevertheless, the cyclic ether pathway appeared to be highly influenced by the incubation conditions. Under anaerobic conditions, the peak abundances of **M8** and **M9** increased by approximately 300–400% (Figure 5). In contrast, **M7** showed an approximately 60% decrease in peak abundance under anaerobic conditions. However, this decrease may not truly reflect the actual change, as the significant deviation within the D6 samples could have affected the result. Overall, the increase in abundance under anaerobic conditions suggests that this pathway is more favored under anaerobic conditions.

The current results clearly demonstrate the osteogenic effects of SECO and the microbial transformation products in terms of the osteoblastic cell viability, cell differentiation, and gene expression of the relevant bone markers. More importantly, the effective concentrations of metabolites such as **M2**, **M8**, **M9**, enterodiol, and enterolactone were as low as 10^−^^12^ M, a finding which has not been reported for other microbial transformed products. Nevertheless, an in vitro study showed that low concentrations of enterodiol (0.1 mg/mL) and enterolactone (0.01 mg/°C) were able to increase the ALP activities of MG-63 cells [50]. However, the concentrations were much higher than the levels normally detected in serum. Osteoprotogerin (OPG) and the receptor activator of nuclear factor kappa-B ligand (RANKL) are a pair of factors involved in the interaction between osteoblasts and osteoclasts. OPG serves as a decoy receptor for RANKL and disturbs its interactions with RANK, thus inhibiting RANKL-induced osteoclastogenesis and bone resorption [51]. The effects of phytoestrogen on bone are believed to be mediated by estrogen receptors (ERs) via genomic pathways in which compounds bind to ERs and regulate the transcription of osteogenic factors like ALP, OPG, and RANKL [39,52]. It has been suggested that the binding affinities of microbial-transformed enterodiol and enterolactone towards ER are more potent than those of precursor lignans [27]. It is possible that the identified metabolites might exert osteogenic effects through an estrogen-dependent pathway. In addition, it seems that SECO may assert certain beneficial effects on bone health even without the presence of gut microflora. However, the presence of the gut microflora certainly converted them into a form that is more potent and more available in the body.

The results of microbial incubation showed that enterodiol, enterolactone, and **M9**, i.e., the enterolignans confirmed with potential bone-protective effects, appeared in the microbial culture after in vitro fermentation for 2–3 days and remained in relatively high abundance for multiple days. In line with the current findings, a pharmacokinetic study on postmenopausal women showed that following oral intake, secoisolariciresinol-diglycoside hydrolyzed to SECO very quickly, and enterolactone began to appear in the circulation within 24 h and peaked at 36 h. A significant amount of enterolactone was still circulating in the body after 60 h [34]. This long appearance time allows the end products to be sustained in the circulation for a longer time to exert beneficial effects. It is possible that the incubation conditions and the microbiota composition may not be exactly the same in vivo, but in vitro fermentation can reflect what would happen inside the gut.

In summary, the present study revealed two metabolic pathways of the microbial biotransformation of SECO, in which nine metabolites were identified. In addition, all the identified metabolites were obtained via total synthesis or purchase. Moreover, enterodiol and enterolactone, the end products of the cyclic ether pathway **M9**, exerted more potent osteogenic effects than their lignan precursor. Thus, the presence of the gut microbiota would at least enhance the beneficial effects of dietary lignans on bone remodeling. The current work provides a useful platform for understanding the microbial transformation of natural products. Furthermore, the current study improves our understanding of the microbial transformation of SECO and provides new approaches and candidates for osteoporosis treatment.

## 4. Materials and Methods

### 4.1. Chemicals

All the chemicals were purchased from Sigma-Aldrich (St. Louis, MO, USA) unless otherwise stated. Enterodiol was purchased from Ost Research Chemicals Inc. (San Francisco, CA, USA). Matairesinol was purchased from Kewelchem (Shanghai, China), and 4, 4′-(((3*R*, 4*R*)-tetrahydrofuran-3, 4-diyl)bis(methylene))bis(2-methoxyphenol) was purchased from the National Institutes for Food and Drug Control (Beijing, China). The stock solution of SECO for microbial transformation was prepared in dimethyl sulfoxide (DMSO) at the concentration of 200 mg/mL. The stock solution of SECO for LCMS detection was prepared in methanol at the concentration of 1 mg/mL. Stock solutions of SECO and the proposed metabolites for the cell experiments were prepared in ethanol at the concentration of 10 mM.

### 4.2. Human Fecal Sample Collection and Preparation

Fecal samples were freshly collected from four healthy volunteers (two men and two women, aged 25–40 years) who were non-smokers and were not taking any probiotics or antibiotics for at least two weeks prior to donation. The fecal samples were mixed with anaerobic buffer solution containing 8.8 g/L K_2_HPO_4_, 6.8 g/L KH_2_PO_4_, 0.1 g/L sodium thioglycolate, and 15 mg/L sodium dithionite (10% *w*/*v*) and homogenized with a stomacher (Seward, AK, USA) at 200 rpm for 5 min. The filtrate-containing bacteria were then aliquoted and stored at −80 °C until further use.

### 4.3. Microbial Transformation of SECO

The aliquoted fecal solution was inoculated into fresh sterile brain heart infusion (BHI) broth (Merck Millipore, Burlington, MA, USA) containing 0.5 mg/mL SECO in a ratio of 1:100 (*v*/*v*). The bacterial culture was incubated at 37 °C under anaerobic (0% oxygen, 10% carbon dioxide, and 5% hydrogen in nitrogen) or microaerobic (5% oxygen, 10% carbon dioxide, and 5% hydrogen in nitrogen) conditions in an anaerobic chamber (Baker Ruskinn Concept 400 M). The samples were periodically collected from the bacterial culture at 0, 8, and 24 h, as well as on days 2, 3, 4, 5, 6, and 7 after inoculation, and were stored at −80 °C until the liquid chromatography–mass spectrometry (LC-MS) analysis. Three parallel cultures were run for each time point. The samples were extracted with methanol through vortexing for 60 s at 4 °C 3 times. After drying with N_2_, the residue was resuspended in a 100 μL mixture of methanol and water (95:5, *v*/*v*).

### 4.4. Ultra-High-Performance Liquid Chromatography–Orbitrap–MS/MS Analysis

A 3µL aliquot sample was injected into a Waters ACQUITY UPLC system (Waters Corp., Milford, MA, USA) and separated using Waters ACQUITY UPLC HSS T3 Column (1.8 μm, 2.1 mm × 100 mm) with HSS T3 pre-column (1.8 μm, 2.1 mm × 5 mm, Waters Corporation, Milford, MA). The mobile phase consisted of combinations of A (0.1% formic acid in water, *v*/*v*) and B (0.1% formic acid in acetonitrile, *v*/*v*) at a flow rate of 0.30 mL/min with an elution gradient, as follows: 0–1 min 5% B; 1–1.5 min 35% B, 1.5–3 min 50% B, 3–6.5 min 55% B, 6.5–8.5 min 95% B, 8.5–10.5 min 95% B, 10.5–11 min 95%, and 11–14 min 95% B. A 3 min post-run time was set to ensure full equilibration, while the column and sample chamber temperatures were 40 °C and 4 °C, respectively.

Mass analysis was performed using a Thermo Orbitrap Fusion Lumos Tribrid Mass Spectrometry system (Thermo Fisher Scientific, Waltham, MA, USA) equipped with a heated electrospray ion source (H-ESI) operating in both the positive (+ESI) and negative (−ESI) ion modes. The H-ESI parameters included a spray voltage, 3500 V for the positive ESI and 2300 V for the negative ESI; Nitrogen gas (≥99.999%) was used as sheath gas, nebulizer auxiliary gas and sweep gas with flow rate 35, 10 and 3 arbitrary units respectively. The ion transfer tube temperature and vaporizer temperature were set at 300 °C and 320 °C, respectively. A full-scan MS analysis was conducted within the mass range of 70–1200 *m*/*z* at a resolution of 120,000, with an automatic gain control (AGC) target of 2.0 × 105 and a maximum injection time of 50 ms.

### 4.5. Data Processing and Metabolite Identification

The UPLC–orbitrap–MS data obtained in both the positive and negative ion modes were initially processed using Progenesis QI (version 2.3; Nonlinear Dynamics, Edmonton, AB, Canada) for peak picking and peak alignment. Subsequently, the data were normalized to the total peak area of each sample. The normalized data were then filtered to remove unstable signals, with a coefficient of variation (CV%) > 30% across the QC samples. A multi-variance analysis was conducted using the Extended Statistical tool (EZinfo v2.0 software, Umetrics AB, Umeå, Sweden). All the samples were UV-scaled for principal component analysis (PCA) and Partial Least Squares Discriminant Analysis (PLS-DA). Metabolites with a high variable important projection (VIP) value in the PLS-DA were considered as strongly contributing to the model and were selected for further analysis. Therefore, the variable importance projection (VIP) value, reasonable biotransformation patterns, and MS2 spectra were used as three parameters to predict the intermediates in SECO metabolism.

### 4.6. Synthesis of Identified Metabolites

**Figure 8:**
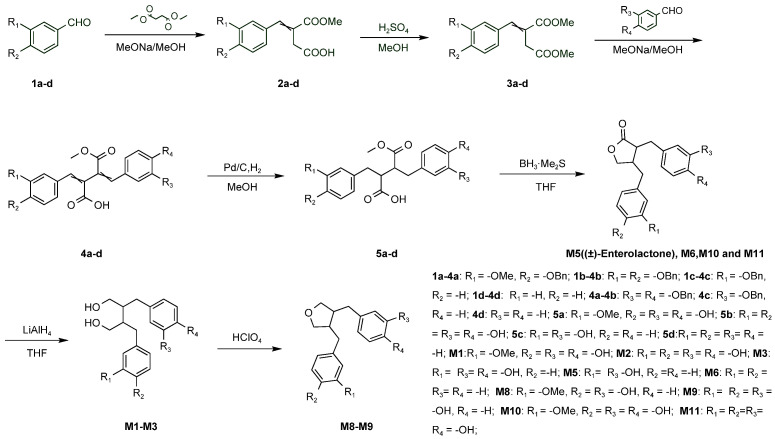
Synthesis scheme of lignan metabolites.

Column chromatography was carried out on silica gel (200–300 mesh) manufactured by Qingdao Haiyang Chemical Group Co., Ltd., Qingdao, China. Analytical TLC was performed on silica gel plates and visualized under ultraviolet light (254 nm). ^1^H NMR spectra were recorded on a Bruker spectrometer with 400 MHz for proton (^1^H NMR). LCMS (ESI) was recorded on an Agilent 6125B mass spectrometer coupled with an Agilent 1260 Infinity LC system. The synthesis of the 8 compounds is shown in Figure 8.

*4-(4-(Benzyloxy)-3-methoxyphenyl)-3-(methoxycarbonyl)but-3-enoic acid (***2a***)*. To a stirred solution of aldehyde 1a (5 g, 20.64 mmol) in MeOH (20 mL), we added diethyl succinate (3.96 g, 22.69 mmol) and MeONa/MeOH solution (2.45 g, 45.40 mmol, 30%). The reaction mixture was stirred at 70 °C for 6 h. Then, the mixture was poured into ice water and adjusted to pH 4 with 4 N HCl. The mixture was extracted with ethyl acetate (50 mL × 3). The combined organic layers were washed with brine and dried over anhydrous Na_2_SO_4_. After filtration, the solvent was removed under vacuum and the residue was purified using silica gel column chromatography (n-heptane/ethyl acetate = 4/1~2/1) to afford compound **2a** (3.15 g, 43%) as an orange-yellow oil.

*Dimethyl 2-(4-(benzyloxy)-3-methoxybenzylidene)succinate (***3a***).* To a stirred solution of compound **2a** (3 g, 8.42 mmol) in MeOH (12 mL), we added concentrated H_2_SO_4_ (0.92 mL, 16.84 mmol). The reaction mixture was stirred at 70 °C for 3 h. The mixture was cooled to 0 °C in an ice bath and was neutralized with saturated NaHCO_3_ to approximately pH 7. The mixture was extracted with ethyl acetate (25 mL × 3). The combined organic layers were washed with brine and dried over anhydrous Na_2_SO_4_. After filtration, the solvent was removed under vacuum to afford compound **3a** (2.9 g, 93%) as a yellow oil.

*2-(4-(Benzyloxy)-3-methoxybenzylidene)-4-(3,4-bis(benzyloxy)phenyl)-3-(methoxycarbonyl)but-3-enoic acid (***4a***).* To a stirred solution of aldehyde 2 (1.72 g, 5.4 mmol) in MeOH (8 mL), we added compound **3a** (2 g, 5.4 mmol) and MeONa/MeOH solution (0.58 g, 10.8 mmol, 30%). The reaction mixture was stirred at 70 °C for 6 h. Then, the mixture was poured into ice water and adjusted to pH 4 with 4 N HCl. The mixture was extracted with ethyl acetate (20 mL × 3). The combined organic layers were washed with brine and dried over anhydrous Na_2_SO_4_. After filtration, the solvent was removed under vacuum, and the residue was purified using silica gel column chromatography (n-heptane/ethyl acetate = 5/1 to dichloromethane/methanol = 20/1) to afford compound **4a** (2.3 g, 65%) as an orange-yellow solid.

*3-(3,4-Dihydroxybenzyl)-2-(4-hydroxy-3-methoxybenzyl)-4-methoxy-4-oxobutanoic acid (***5a***).* To a stirred solution of compound **4a** (0.5 g, 0.76 mmol) in MeOH (5 mL), we added Pd/C (0.05 g, 10%, wet). The reaction flask was flushed several times with hydrogen, and then the mixture was stirred at room temperature overnight in a hydrogen atmosphere. The reaction mixture was filtered over celite, and the solvent was removed under vacuum to yield compound **5a** (0.3 g, 99%).

*3-(3,4-Dihydroxybenzyl)-4-(4-hydroxy-3-methoxybenzyl)dihydrofuran-2(3H)-one (***M10***).* To a stirred solution of compound **5a** (0.3 g, 0.77 mmol) in anhydrous THF (9 mL), BH_3_∙Me_2_S (0.5 mL, 4.62 mmol, 10 mol/L) was added at 0 °C in an ice bath. The reaction flask was evacuated and flushed with nitrogen, and the mixture was stirred at room temperature for 1 h in a N_2_ atmosphere. The mixture was quenched with 4 N HCl at 0 °C. Then, the resulting mixture was extracted with ethyl acetate (30 mL × 3). The combined organic layers were washed with brine and dried over anhydrous Na_2_SO_4_. After filtration, the solvent was removed under vacuum, and the residue was purified using prep-HPLC (acetonitrile/water, flow rate: 16 mL/min, concentration: 190 mg/mL, sample size: 0.1 mL) to afford M10 (60 mg, 22%) as a white solid. ^1^H NMR (400 MHz, methanol-*d*_4_) δ 6.72–6.62 (m, 3H), 6.57–6.49 (m 3H), 4.09 (dd, *J* = 8.9, 6.9 Hz, 1H), 3.90 (t, *J* = 8.2 Hz, 1H), 3.80 (s, 3H), 2.86–2.77 (m, 2H), 2.66–2.40 (m, 4H). LCMS: *m*/*z* [M + H]^+^: 345.1.

Compounds **M11**, **M6**, *and (±)- enterolactone* were prepared in a similar manner as described for compound **M10**. *2,3-bis(3,4-dihydroxybenzyl)butyrolactone (***M11***)*: white solid, 35% yield. ^1^H NMR (400 MHz, methanol-*d*_4_) δ 6.77–6.55 (m, 3H), 6.52–6.40 (m, 2H), 6.42–6.39 (m, 1H), 4.08–4.03 (m, 1H), 3.89–3.85 (m, 1H), 3.32–3.10 (m, 1H), 3.09–3.04 (m, 1H), 2.87–2.80 (m, 1H), 2.67–2.56 (m, 1H), 2.55–2.44 (m, 1H), 2.40–2.37 (m, 1H). LCMS: *m*/*z* [M − H]^−^ 329.0; *3,4-dibenzyldihydrofuran-2(3H)-one (M6)*: white solid, 40% yield. ^1^H NMR (400 MHz, methanol-*d*_4_) δ 7.29–7.02 (m, 10H), 3.58–3.50 (m, 4H), 2.80–2.67 (m, 4H). The LCMS had no response; *(±)- enterolactone*: white solid, 30% yield. ^1^H NMR (400 MHz, Chloroform-d) δ 7.20–7.14 (m, 2H), 6.75–6.73 (m, 3H), 6.63–6.56 (m, 2H), 6.52–6.47 (m, 1H), 4.13–4.09 (m, 1H), 3.87–3.83 (m, 1H), 2.96–2.89 (m, 2H), 2.88–2.60 (m, 2H), 2.58–2.46 (m, 2H). LCMS: *m*/*z* [M − H]^−^ 297.0.

*4,4‣-(2,3-Bis(hydroxymethyl)butane-1,4-diyl)bis(benzene-1,2-diol) (***M2***)*. To a stirred solution of **M11** (1.9 g, 5.75 mmol) in anhydrous THF (100 mL), we added LiAlH_4_ (0.43 g, 11.5 mmol) in portions at 0 °C to an ice bath. The reaction flask was evacuated and flushed with N_2_ three times. The mixture was stirred at 0 °C for 2 h in a N_2_ atmosphere. The mixture was quenched with 4 N HCl at 0 °C. Then, the resulting mixture was extracted with ethyl acetate (100 mL × 3). The combined organic layers were washed with brine and dried over anhydrous Na_2_SO_4_. After filtration, the solvent was removed under vacuum, and the residue was purified using silica gel column chromatography (n-heptane/ethyl acetate = 1/1 to dichloromethane/methanol = 10/1) to afford compound **M2** (750 mg, 40% yield) as a white solid. ^1^H NMR (400 MHz, DMSO-*d*_6_) δ 8.59 (d, *J* = 30.7 Hz, 4H), 6.67–6.53 (m, 4H), 6.38–6.35 (m, 2H), 4.61–4.40 (m, 2H), 3.25–3.17 (m, 4H), 2.48–2.27 (m, 4H), 1.80 (d, *J* = 10.9 Hz, 2H). LCMS: *m*/*z* [M − H]^−^: 333.0.

Compounds **M1** and **M3** were prepared in a similar manner as described for compound **M2**. *4-(4-Hydroxy-3-(4-hydroxy-3-methoxybenzyl)-2-(hydroxymethyl)butyl)benzene-1,2-diol (***M1***)*: white solid, 5% yield. ^1^H NMR (400 MHz, DMSO-*d*_6_) δ 8.63 (s, 3H), 6.66–6.48 (m, 5H), 6.40–6.36 (m, 1H), 4.49 (s, 2H), 3.70 (s, 3H), 3.49–3.34 (m, 4H), 2.47–2.36 (m, 4H), 2.02–1.86 (m, 2H). LCMS: *m*/*z* [M − H]^−^: 347.0; *4-(4-hydroxy-3-(3-hydroxybenzyl)-2-(hydroxymethyl)butyl)benzene-1,2-diol (M3)*: white solid. 37% yield. ^1^H NMR (400 MHz, methanol-*d*_4_) δ 7.06–7.05 (m, 1H), 6.82–6.48 (m, 6H), 3.66–3.44 (m, 4H), 2.70–2.48 (m, 4H), 2.09–1.88 (m, 2H). LCMS: *m*/*z* [M − H]^−^: 317.0.

*4-((4-(3-Hydroxybenzyl)tetrahydrofuran-3-yl)methyl)-2-methoxyphenol (***M8***)*. To a stirred of solution M1 (50 mg, 0.15 mmol, mixture) in acetone (5 mL), we added a drop of HClO_4_ (141 μL, 0.015 mmol) with a syringe. The reaction mixture was refluxed at 60 °C for 0.5 h. Afterwards, the mixute was cooled to room temperature and neutralized with a saturated NaHCO_3_ solution to approximately pH 7. The mixture was extracted with ethyl acetate (15 mL × 3). The combined organic layers were washed with brine and dried over anhydrous Na_2_SO_4_. After filtration, the solvent was removed under vacuum, and the residue was purified using prep-HPLC (acetonitrile/water, flow rate: 16 mL/min, concentration: 25 mg/mL, sample size: 0.2 mL) to afford **M8** (50 mg, 7%) as a white solid. ^1^H NMR (400 MHz, methanol-*d*_4_) δ 7.13–7.01 (m, 1H), 6.72–6.58 (m, 6H), 3.85–3.80 (m, 5H), 3.78–3.50 (m, 2H), 2.85 (d, *J* = 12.8 Hz, 1H), 2.59–2.54 (m, 4H), 2.47–2.19 (m, 1H). LCMS: *m*/*z* [M − H]^−^: 313.0.

*4-((4-(3-Hydroxybenzyl)tetrahydrofuran-3-yl)methyl)benzene-1,2-diol (***M9***)* was prepared in a similar manner as **M8**. White solid, 15% yield. ^1^H NMR (400 MHz, DMSO-*d*_6_) δ 6.29–6.25 (m, 1H), 5.92–5.69 (m, 6H), 3.09–2.99 (m, 1H), 2.99–2.91 (m, 1H), 2.82–2.79 (m, 1H), 2.73–2.65 (m, 1H), 2.07–2.95 (m, 1H), 1.85–1.59 (m, 4H), 1.42–1.59 (m, 1H). LCMS: *m*/*z* [M − H]^−^: 299.0.

### 4.7. Culture of MC3T3-E1 and MG-63 Cells

The murine pre-osteoblast MC3T3-E1 cells (Subclone4, CRL-2593) and human osteosarcoma MG-63 cells (CRL-1427TM) were obtained from the American Type Culture Collection (ATCC, Manassas, VA, USA) and maintained in alpha Minimum Essential Medium (α-MEM, Gibco, Grand Island, NY, USA) Dulbecco’s Modified Eagle Medium (DMEM, Gibco, New York, NY, USA), respectively. The culture medium was supplemented with 10% heat-inactivated fetal bovine serum (FBS, Hyclone, Logan, UT, USA) and 100 U/mL penicillin-streptomycin (Gibco, USA). The cells were incubated in an atmosphere of 95% humidity and 5% CO_2_ at 37 °C.

### 4.8. Cell Availability Assay

MC3T3-E1 cells were seeded in 96-well plates until they reached 80% confluence. The cells were treated with the vehicle, 17β-estradiol (E_2_, 10^−^^8^ M), and synthesized compounds (10^−^^12^–10^−^^7^ M) in phenol red-free α-MEM supplemented with 1% charcoal-striped FBS (Gibco, USA) for 24 h. The cell viability was measured using standard MTS (3-(4,5-dimethylthiazol-2-yl)-5-(3-carboxymethoxyphenyl)-2-(4-sulfophenyl)-2H-tetrazolium) assay.

### 4.9. Alkaline Phosphatase (ALP) Activity Assay

MC3T3-E1 cells were seeded in 24-well plates until they reached 80% confluence. To differentiate MC3T3-E1 into mature osteoblasts, the culture medium was replaced with osteogenic medium composed of phenol red-free α-MEM supplemented with 1% charcoal-striped FBS (Gibco, USA), 10 mM β-glycerophosphate, and 50 μg/mL L-ascorbic acid. The cells were treated with the vehicle, 17β-estradiol (10^−^^8^ M), and synthesized compounds (10^−^^12^–10^−^^7^ M) in an osteogenic medium for continuous 7 days. The medium and treatment were renewed every 3 days. The treated cells were lysed with passive lysis buffer (Promega, Madison, NY, USA). ALP activity was determined with a LabAssay ALP kit (Wako, Japan). ALP activity was normalized according to the total protein content, determined via BCA protein assay.

### 4.10. Real-Time Quantitative RT- PCR Analysis

MG-63 cells were treated with the vehicle, 17β-estradiol (10^−^^8^ M), and synthesized compounds (10^−^^12^–10^−^^7^ M) in phenol red-free DMEM supplemented with 1% charcoal-striped FBS (Gibco, USA) for 48 h. To investigate the expression levels of the receptor activator of the NF-κB ligand (RANKL) and osteoprotegerin (OPG), total RNA was isolated using TRIzol reagent (Invitrogen, Carlsbad, CA, USA). After reverse transcription using PrimeScriptTM RT Master Mix (TaKaRa, Kusatsu, Japan), 500 ng of cDNA product was added to the PCR reaction mixture containing TB Green Premix Ex Taq II (TaKaRa, Japan). Real-time PCR was performed with a 7900HT Fast Real-Time PCR System (Applied Biosystems, Carlsbad, CA, USA), with the following amplification conditions and procedures: initial denaturation at 95 °C for 30 s, with 40 cycles of denaturation at 95 °C for 1 s and 60 °C for 20 s. The sequences of the primers for the target gene and the housekeeping gene glyceraldehyde-3-phosphate dehydrogenase (GAPDH) were as follows: OPG forward 5′-ACAGCAAAGTGGAAGACCGT-3′, reverse 5′-CCTTCCTTGCATTCGCACAC-3′; RANKL forward 5′-GGGGAAAACTTGCAGCTAAGG-3′, reverse 5′-AATTTGCGGCACTTGTGGAA-3′; and GAPDH forward 5′-ACCCACTCCTCCACCTTTGAC-3′, reverse 5′-TGTTGCTGTAGCCAAATTCGTT-3′. Relative gene expression was calculated using the 2^−ΔΔCT^ method.

### 4.11. Statistical Analysis

Data are presented as the mean ± standard error of mean (SEM). Intergroup differences between different treatments were analyzed via one-way analysis of variance (ANOVA) followed by Tukey’s post hoc test for multiple comparisons (GraphPad Prism 8.0, San Diego, CA, USA). A *p* value of less than 0.05 was considered statistically significant.

## 5. Conclusions

The presence of the gut microbiota could offer a good way to form diverse enterolignans with bone-protective effects. The current study improves our understanding of the microbial transformation of SECO and provides new approaches and candidates for osteoporosis treatment.

## Figures and Tables

**Table 1:** Metabolites identified after the biotransformation of SECO in human gut microbiota culture.

Metabolites	Formula	+ESI				−ESI			
		Rt (min)	*m*/*z*	Adduct	MS2 Fragments (*m/z*)	Rt (min)	*m*/*z*	Adduct	MS2 Fragments (*m*/*z*)
SECO	C_20_H_26_O_6_	4.49	345.169	[M − H_2_O + H]^+^	295.1319, 263.1059, 235.1111, 189.0904, 137.0593	4.49	361.1657	[M − H]^−^	209.0815, 179.0705, 165.0549, 136.0520, 122.0364
**M1**	C_19_H_24_O_6_	4.25	313.143	[M − 2H_2_O + H]^+^	295.1332, 263.1066, 235.1113, 189.0905, 149.0592, 137.0435	4.24	347.1500	[M − H]^−^	209.0799, 179.0694, 165.0542, 136.0525, 122.0360
**M2**	C_18_H_22_O_6_	4.04	299.127	[M − 2H_2_O + H]^+^	263.1064, 235.1115, 149.0590	4.04	315.1238	[M − H_2_O − H]^−^	122.0360
**M3**	C_18_H_22_O_5_	4.33	283.133	[M − 2H_2_O + H]^+^	189.0905, 137.0589	4.33	317.1394	[M − H]^−^	122.0369, 107.0484
**M4** (Enterodiol)	C_18_H_22_O_4_	4.68	303.159	[M + H]^+^	159.0800, 133.0644, 107.0489	4.69	301.1445	[M − H]^−^	107.0494
**M5** (Enterolactone)	C_18_H_18_O_4_	5.40	299.127	[M + H]^+^	263.1059, 235.1108, 133.0640, 107.0484	5.40	297.1132	[M − H]^−^	165.0549, 107.0494
**M6**	C_18_H_18_O_2_	4.70	267.138	[M + H]^+^	N.D	N.D	N.D	N.D	N.D
**M7**	C_20_H_24_O_6_	N.D	N.D	N.D	N.D	5.24	343.1551	[M − H]^−^	N.D
**M8**	C_19_H_21_O_5_	4.62	297.149	[M − 2H_2_O + H]^+^	N.D	N.D	N.D	N.D	N.D
**M9**	C_18_H_20_O_4_	4.95	265.123	[M − 2H_2_O + H]^+^	263.1052, 133.0639, 159.0704, 107.0489	4.95	299.1289	[M − H]^−^	107.0495

N.D, not detected.

**Table 2:** Effects of identified metabolites on the gene expression of bone markers.

Metabolites	OPG	RANKL	OPG/RANKL
**M1**	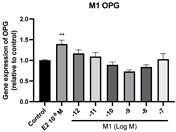	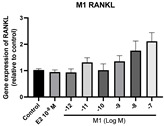	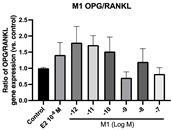
**M2**	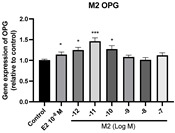	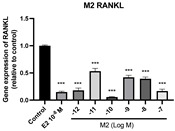	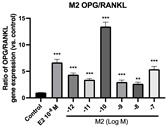
**M3**	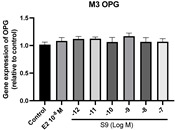	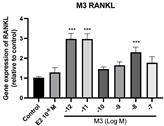	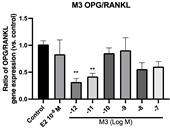
**M4** (Enterodiol)	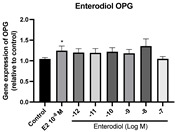	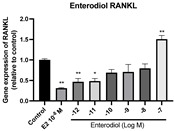	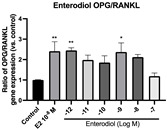
**M5** (Enterolactone)	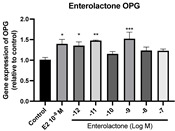	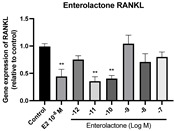	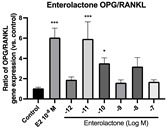
**M6**	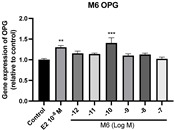	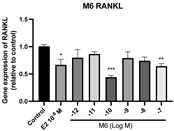	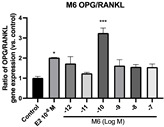
**M7**	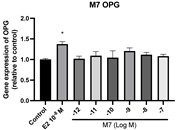	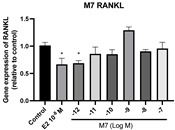	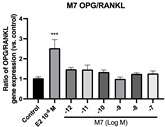
**M8**	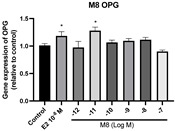	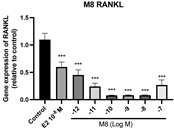	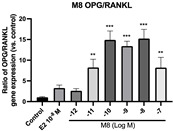
**M9**	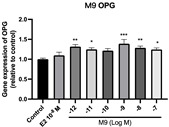	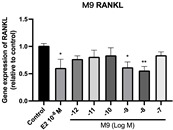	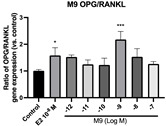

Expressions of bone markers were measured via RT-PCR. Data are shown as mean ± SEM and analyzed using one-way ANOVA, followed by Tukey’s multiple comparison test. * *p* < 0.05, ** *p* < 0.01, *** *p* < 0.001 vs. control (*n* = 6).

## Data Availability

Not applicable.

## Sample Availability

Samples of the compounds are available from the first and corresponding authors.

## References

[1] Lorentzon M., Johansson H., Harvey N., Liu E., Vandenput L., McCloskey E., Kanis J. (2022). Osteoporosis and fractures in women: The burden of disease. Climacteric.

[2] Bowring C., Francis R. (2011). National Osteoporosis Society’s Position statement on hormone replacement therapy in the prevention and treatment of osteoporosis. Menopause Int..

[3] Langer R., Hodis H., Lobo R., Allison M. (2021). Hormone replacement therapy–where are we now?. Climacteric.

[4] Cagnacci A., Venier M. (2019). The controversial history of hormone replacement therapy. Medicina.

[5] Ranich T., Bhathena S.J., Velasquez M.T. (2001). Protective effects of dietary phytoestrogens in chronic renal disease. J. Ren. Nutr..

[6] Adlercreutz H., Höckerstedt K., Bannwart C., Bloigu S., Hämäläinen E., Fotsis T., Ollus A. (1987). Effect of dietary components, including lignans and phytoestrogens, on enterohepatic circulation and liver metabolism of estrogens and on sex hormone binding globulin (SHBG). J. Steroid Biochem..

[7] Landete Iranzo J.M. (2012). Plant and mammalian lignans: A review of source, intake, metabolism, intestinal bacteria and health. Food Res. Int..

[8] Mazur W. (1998). 11 Phytoestrogen content in foods. Baillière’s Clin. Endocrinol. Metab..

[9] Kitts D., Yuan Y., Wijewickreme A., Thompson L. (1999). Antioxidant activity of the flaxseed lignan secoisolariciresinol diglycoside and its mammalian lignan metabolites enterodiol and enterolactone. Mol. Cell. Biochem..

[10] Cheng C., Yu X., McClements D.J., Huang Q., Tang H., Yu K., Xia X., Chen P., Wang X.T., Deng Q.C. (2019). Effect of flaxseed polyphenols on physical stability and oxidative stability of flaxseed oil-in-water nanoemulsions. Food Chem..

[11] Murkies A., Wilcox G., Davis S. (1998). Phytoestrogens-review. J. Clin. Endocrinol. Metab..

[12] Thompson L.U. (1998). Experimental studies on lignans and cancer. Bailliere’s Clin. Endocrinol. Metab..

[13] Asad B., Khan T., Gul F.Z., Ullah M.A., Drouet S., Mikac S., Garros L., Ferrier M., Bose S., Munsch T. (2021). Scarlet flax *Linum grandiflorum* (L.) in vitro cultures as a new source of antioxidant and anti-inflammatory lignans. Molecules.

[14] Adlercreutz H., Mazur W., Bartels P., Elomaa V.-V., Watanabe S., Wahala K., LandstoM M., Lundin E., Bergh A., Damber J.E. (2000). Phytoestrogens and prostate disease. J. Nutr..

[15] Kajla P., Sharma A., Sood D.R. (2015). Flaxseed—A potential functional food source. J. Food Sci. Technol..

[16] Bloedon L.T., Balikai S., Chittams J., Cunnane S.C., Berlin J.A., Rader D.J., Szapary P.O. (2008). Flaxseed and cardiovascular risk factors: Results from a double blind, randomized, controlled clinical trial. J. Am. Coll. Nutr..

[17] Imran M., Ahmad N., Anjum F.M., Khan M.K., Mushtaq Z., Nadeem M., Hussain S. (2015). Potential protective properties of flax lignan secoisolariciresinol diglucoside. Nutr. J..

[18] Hafiz H., Commane D., Walton G., Jackson K. (2022). Effect of Whole Flaxseed on Bone Turnover Markers and Gut Microbiota in Menopausal-Related Bone Loss. Bioactive Compounds from Multifarious Natural Foods for Human Health.

[19] Xiao H.H., Chan C., Wong K., Kam-wah D., Chan S., Cooper R. (2015). Lignans from *Sambucus williasmii* Hance against osteoporosis: A pharmacodynamic and pharmacokinetic study. Planta Medica.

[20] Xiao H.H., Wong M.S., Yao X.S. (2016). A Lignan-Rich Bioactive Fraction of Sambucus williamsii Hance Exerts Oestrogen-Like Bone Protective Effects in Aged Ovariectomized Rats and Osteoblastic Cells. Nutritional Influences on Bone Health: 9th International Symposium.

[21] Peñalvo J.L., Nurmi T., Haajanen K., Al-Maharik N., Botting N., Adlercreutz H. (2004). Determination of lignans in human plasma by liquid chromatography with coulometric electrode array detection. Anal. Biochem..

[22] Nurmi T., Voutilainen S., Nyyssönen K., Adlercreutz H., Salonen J.T. (2003). Liquid chromatography method for plant and mammalian lignans in human urine. J. Chromatogr. B.

[23] Bannwart C., Adlercreutz H., Wähälä K., Brunow G., Hase T. (1989). Detection and identification of the plant lignans lariciresinol, isolariciresinol and secoisolariciresinol in human urine. Clin. Chim. Acta.

[24] Setchell K., Lawson A., Mitchell F., Adlercreutz H., Kirk D., Axelson M. (1980). Lignans in man and in animal species. Nature.

[25] Setchell K., Borriello S., Gordon H., Lawson A., Harkness R., Morgan D. (1981). Lignan formation in man—Microbial involvement and possible roles in relation to cancer. Lancet.

[26] Axelson M., Sjövall J., Gustafsson B., Setchell K. (1982). Origin of lignans in mammals and identification of a precursor from plants. Nature.

[27] Mueller S.O., Simon S., Chae K., Metzler M., Korach K.S. (2004). Phytoestrogens and their human metabolites show distinct agonistic and antagonistic properties on estrogen receptor α (ERα) and ERβ in human cells. Toxicol. Sci..

[28] Brooks J.D., Thompson L.U. (2005). Mammalian lignans and genistein decrease the activities of aromatase and 17β-hydroxysteroid dehydrogenase in MCF-7 cells. J. Steroid Biochem. Mol. Biol..

[29] Jacobs M.N., Nolan G.T., Hood S.R. (2005). Lignans, bacteriocides and organochlorine compounds activate the human pregnane X receptor (PXR). Toxicol. Appl. Pharmacol..

[30] Xiao H.H., Sham T.T., Chan C., Li M.H., Chen X., Wu Q.C., Mok D.K.W., Yao X.S., Wong M.S. (2018). A metabolomics study on the bone protective effects of a lignan-rich fraction from *Sambucus williamsii* Ramulus in aged rats. Front. Pharmacol..

[31] Xiao H.H., Dai Y., Wan H.Y., Wong M.S., Yao X.S. (2011). Bone-protective effects of bioactive fractions and ingredients in Sambucus williamsii HANCE. Br. J. Nutr..

[32] Xiao H.H., Zhu Y.X., Lu L., Zhou L.P., Poon C.C., Chan C.O., Wang L.J., Cao S., Yu W.X., Wong K.Y. (2022). The Lignan-Rich Fraction from *Sambucus williamsii* Hance Exerts Bone Protective Effects via Altering Circulating Serotonin and Gut Microbiota in Rats. Nutrients.

[33] Quartieri A., García-Villalba R., Amaretti A., Raimondi S., Leonardi A., Rossi M. (2016). Detection of novel metabolites of flaxseed lignans in vitro and in vivo. Mol. Nutr. Food Res..

[34] Setchell K.D., Brown N.M., Zimmer-Nechemias L., Wolfe B., Jha P., Heubi J.E. (2014). Metabolism of secoisolariciresinol-diglycoside the dietary precursor to the intestinally derived lignan enterolactone in humans. Food Funct..

[35] Niemeyer H.B., Honig D.M., Kulling S.E., Metzler M. (2003). Studies on the metabolism of the plant lignans secoisolariciresinol and matairesinol. J. Agric. Food Chem..

[36] Wishart D.S., Tian S., Allen D., Oler E., Peters H., Lui V.W. (2022). BioTransformer 3.0—A web server for accurately predicting metabolic transformation products. Nucleic Acids Res..

[37] Senizza A., Rocchetti G., Mosele J.I., Patrone V., Callegari M.L., Morelli L. (2020). Lignans and gut microbiota: An interplay revealing potential health implications. Molecules.

[38] Orimo H. (2010). The mechanism of mineralization and the role of alkaline phosphatase in health and disease. J. Nippon. Med. Sch..

[39] Ho M.X., Poon C.C.W., Wong K.C., Qiu Z.C., Wong M.S. (2018). Icariin, but not genistein, exerts osteogenic and anti-apoptotic effects in osteoblastic cells by selective activation of non-genomic ERα signaling. Front. Pharmacol..

[40] Tham D.M., Gardner C.D., Haskell W.L. (1998). Potential health benefits of dietary phytoestrogens: A review of the clinical, epidemiological, and mechanistic evidence. J. Clin. Endocrinol. Metab..

[41] Wang L.Q., Meselhy M.R., Li Y., Qin G.W., Hattori M. (2000). Human intestinal bacteria capable of transforming secoisolariciresinol diglucoside to mammalian lignans, enterodiol and enterolactone. Chem. Pharm. Bull..

[42] Clavel T., Henderson G., Engst W., Dore J., Blaut M. (2006). Phylogeny of human intestinal bacteria that activate the dietary lignan secoisolariciresinol diglucoside. Fems Microbiol. Ecol..

[43] Clavel T., Lippman R., Gavini F., Dore J., Blaut M. (2007). Clostridium saccharogumia sp nov and Lactonifactor longoviformis gen. nov., sp nov., two novel human faecal bacteria involved in the conversion of the dietary phytoestrogen secoisolariciresinol diglucoside. Syst. Appl. Microbiol..

[44] Jin J.S., Kakiuchi J., Hattori M. (2007). Enantioselective oxidation of enterodiol to enterolactone by human intestinal bacteria. Biol. Pharm. Bull..

[45] Struijs K., Vincken J.P., Gruppen H. (2009). Bacterial conversion of secoisolariciresinol and anhydrosecoisolariciresinol. J. Appl. Microbiol..

[46] Jin J.S., Zhao Y.F., Nakamura N., Akao T., Kakiuchi N., Min B.S. (2007). Enantioselective dehydroxylation of enterodiol and enterolactone precursors by human intestinal bacteria. Biol. Pharm. Bull..

[47] Corona G., Kreimes A., Barone M., Turroni S., Brigidi P., Keleszade E. (2020). Impact of lignans in oilseed mix on gut microbiome composition and enterolignan production in younger healthy and premenopausal women: An in vitro pilot study. Microb. Cell Factories.

[48] Peiroten A., Gaya P., Alvarez I., Bravo D., Landete J.M. (2019). Influence of different lignan compounds on enterolignan production by Bifidobacterium and Lactobacillus strains. Int. J. Food Microbiol..

[49] Bravo D., Peirotén Á., Álvarez I., Landete J.M. (2017). Phytoestrogen metabolism by lactic acid bacteria: Enterolignan production by Lactobacillus salivarius and Lactobacillus gasseri strains. J. Funct. Foods.

[50] Feng J., Shi Z.L., Ye Z.M. (2008). Effects of metabolites of the lignans enterolactone and enterodiol on osteoblastic differentiation of MG-63 cells. Biol. Pharm. Bull..

[51] Boyle W.J., Simonet W.S., Lacey D.L. (2003). Osteoclast differentiation and activation. Nature.

[52] Sun L.J., Li C., Wen X.H., Guo L., Guo Z.F., Liao L.Q. (2021). Icariin stimulates hFOB 1.19 osteoblast proliferation and differentiation via OPG/RANKL mediated by the estrogen receptor. Curr. Pharm. Biotechnol..

